# 2,4,5-Triphenyl-1,3,2-dioxa­phospho­lan-2-one

**DOI:** 10.1107/S1600536811024202

**Published:** 2011-06-25

**Authors:** David B. Cordes, Guoxiong Hua, Alexandra M. Z. Slawin, J. Derek Woollins

**Affiliations:** a School of Chemistry, University of St Andrews, Fife KY16 9ST, Scotland

## Abstract

The dioxaphospho­lane ring in the title compound, C_20_H_17_O_3_P, adopts an envelope conformation about one of the ring carbons. The benzene rings of the compound do not form face-to-face π–π inter­actions, instead weak C—H⋯π inter­actions occur between adjacent mol­ecules. The methine H atoms on the dioxaphospho­lane ring form weak C—H⋯O hydrogen bonds to the oxide group of an adjacent mol­ecule.

## Related literature

For the synthesis of the title compound and isomeric forms, see: Ovchinnikov *et al.* (1979 ▶, 1995 ▶); Chauvin (1990 ▶). For related structures of dioxaphospho­lane oxides, see: Hoppe *et al.* (1985 ▶); Ananikov *et al.* (2010 ▶); Han *et al.* (2008 ▶).
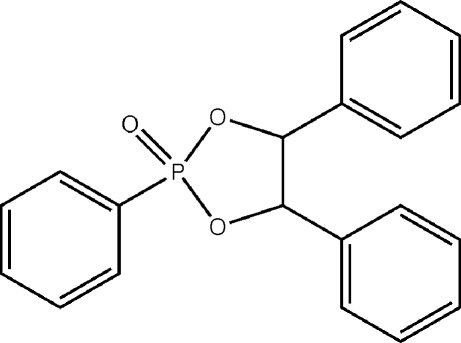


## Experimental

### 

#### Crystal data

C_20_H_17_O_3_P
*M* *_r_* = 336.31
Monoclinic, 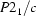

*a* = 16.744 (12) Å
*b* = 6.098 (4) Å
*c* = 17.300 (13) Å
β = 111.810 (15)°
*V* = 1640 (2) Å^3^
*Z* = 4
Mo *K*α radiation
μ = 0.18 mm^−1^
*T* = 93 K
0.20 × 0.01 × 0.01 mm

#### Data collection

Rigaku Mercury CCD diffractometer
Absorption correction: multi-scan (*CrystalClear*; Rigaku, 2010 ▶) *T*_min_ = 0.435, *T*_max_ = 1.000
10244 measured reflections
3462 independent reflections
2034 reflections with *I* > 2σ(*I*)
*R*_int_ = 0.109

#### Refinement

*R*[*F* ^2^ > 2σ(*F* ^2^)] = 0.090
*wR*(*F* ^2^) = 0.256
*S* = 1.01
3462 reflections
217 parameters
H-atom parameters constrained
Δρ_max_ = 0.41 e Å^−3^
Δρ_min_ = −0.53 e Å^−3^

### 

Data collection: *CrystalClear* (Rigaku, 2010 ▶); cell refinement: *CrystalClear*; data reduction: *CrystalClear*; program(s) used to solve structure: *SIR2004* (Burla *et al.*, 2005 ▶); program(s) used to refine structure: *SHELXTL* (Sheldrick, 2008 ▶); molecular graphics: *SHELXTL*; software used to prepare material for publication: *SHELXTL*.

## Supplementary Material

Crystal structure: contains datablock(s) global, I. DOI: 10.1107/S1600536811024202/fj2429sup1.cif

Structure factors: contains datablock(s) I. DOI: 10.1107/S1600536811024202/fj2429Isup2.hkl

Supplementary material file. DOI: 10.1107/S1600536811024202/fj2429Isup3.cml

Additional supplementary materials:  crystallographic information; 3D view; checkCIF report

## Figures and Tables

**Table 1:** Hydrogen-bond geometry (Å, °) *Cg*1 is the centroid of the C15–C20 ring.

*D*—H⋯*A*	*D*—H	H⋯*A*	*D*⋯*A*	*D*—H⋯*A*
C1—H1⋯O3^i^	1.00	2.59	3.248 (5)	123 (3)
C2—H2⋯O3^i^	1.00	2.29	3.115 (5)	139 (3)
C12—H12⋯*Cg*1^ii^	0.95	2.94	3.838 (5)	158 (3)
C12—H12⋯C18^ii^	0.95	2.83	3.585 (6)	138 (3)

Symmetry codes: (i) 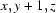; (ii) 
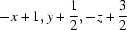
.

**Fig. 1.**
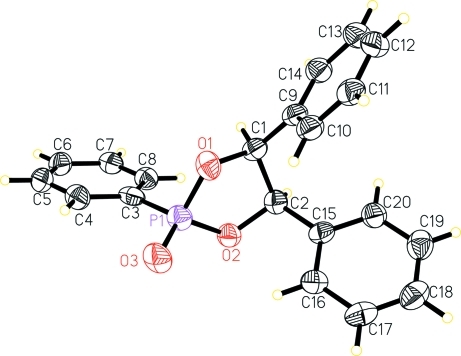
The structure of the title compound with displacement ellipsoids drawn at the 50% probability level.

## supplementary crystallographic information

### Comment

The previously known title compound (Ovchinnikov *et al.*, 1995) has been prepared by the reaction of Woollins' reagent with 1,2-diphenylethane-1,2-diol. The resulting dioxaphospholane ring in the title compound is similar to the only three structurally known phospholanes, bond lengths about P being very similar (P—O, 1.586–1.604 Å, P═O, 1.461–1.467 Å, P—C, 1.765–1.815 Å; Hoppe *et al.*, 1985, Han *et al.*, 2008 and Ananikov *et al.*, 2010). The dioxaphospholane ring displays an envelope conformation about C1, the torsion angles O2—P1—O1—C1 and P1—O2—C2—C1 being 19.0 (2) and -18.8 (3) °, respectively. The phenyl rings in the title compound do not form face-to-face π–π interactions, instead weak CH···π interactions result at a distance of 2.94 (5) Å. The phospholane oxide oxygen forms weak hydrogen bonds with the H1 and H2 H atoms, at distances of 2.29 (4) and 2.59 (4) Å forming chains along the [0 1 0] direction.

### Experimental

A mixture of diphenylethane-1,2-diol (0.214 g, 1.0 mmol) and Woollins' reagent (0.54 g, 1.0 mmol) in 20 ml of dry toluene was stirred at room temperature for 3 h. Then the mixture was heated to 60 °C with stirring for 2 h. The red suspension disappeared and a grayish-green solution formed. Following cooling to room temperature and removal of the solvent *in vacuuo* the residue was purified by silica gel column chromatography (dichloromethane eluent) to give the title compound as a pale green solid in low yield (0.055 g, 14%). Crystals suitable for X-ray structure determination were obtained from the diffusion of hexane into a dichloromethane solution of the title compound. Selected IR (KBr, cm^-1^): 1439(*m*), 1269(*m*), 1133(*m*), 992(*s*), 870(*m*), 840(*m*), 716(*s*), 693(*s*), 510(*m*). ^1^H NMR (CD_2_Cl_2_, δ), 8.06–7.94 (m, 2H, ArH), 7.71–7.55 (m, 2H, ArH), 7.23–7.05 (m, 11H, ArH), 5.88 (m, *J* = 9.1 Hz, 2H, CH) p.p.m.. ^13^C NMR (CD_2_Cl_2_, δ), 134.4, 131.9, 130.9, 130.6, 129.1, 128.4, 128.1, 126.8, 83.9 (COO) p.p.m.. ^31^P NMR (CD_2_Cl_2_, δ), 34.88 p.p.m..

### Refinement

All the crystals chosen appeared to be poorly diffracting at higher angles, with missing independent data in the experimentally measured range. All H atoms were included in calculated positions (C—H distances are 1.00 Å for methine H atoms and 0.95 Å for phenyl H atoms) and refined as riding atoms with *U*_iso_(H) = 1.2 *U*_eq_(parent atom).

### Crystal data

**Table:**

C_20_H_17_O_3_P	*F*(000) = 704
*M**_r_* = 336.31	*D*_x_ = 1.362 Mg m^−^^3^
Monoclinic, *P*2_1_/*c*	Mo *K*α radiation, λ = 0.71073 Å
Hall symbol: -P 2ybc	Cell parameters from 4362 reflections
*a* = 16.744 (12) Å	θ = 2.1–28.5°
*b* = 6.098 (4) Å	µ = 0.18 mm^−^^1^
*c* = 17.300 (13) Å	*T* = 93 K
β = 111.810 (15)°	Needle, colorless
*V* = 1640 (2) Å^3^	0.20 × 0.01 × 0.01 mm
*Z* = 4	

### Data collection

**Table:**

Rigaku Mercury CCD diffractometer	3462 independent reflections
Radiation source: rotating anode	2034 reflections with *I* > 2σ(*I*)
confocal	*R*_int_ = 0.109
Detector resolution: 14.7059 pixels mm^-1^	θ_max_ = 28.6°, θ_min_ = 1.3°
ω and φ scans	*h* = −18→21
Absorption correction: multi-scan (*CrystalClear*; Rigaku, 2010)	*k* = −8→7
*T*_min_ = 0.435, *T*_max_ = 1.000	*l* = −22→19
10244 measured reflections	

### Refinement

**Table:**

Refinement on *F*^2^	Primary atom site location: structure-invariant direct methods
Least-squares matrix: full	Secondary atom site location: difference Fourier map
*R*[*F*^2^ > 2σ(*F*^2^)] = 0.090	Hydrogen site location: inferred from neighbouring sites
*wR*(*F*^2^) = 0.256	H-atom parameters constrained
*S* = 1.01	*w* = 1/[σ^2^(*F*_o_^2^) + (0.1305*P*)^2^] where *P* = (*F*_o_^2^ + 2*F*_c_^2^)/3
3462 reflections	(Δ/σ)_max_ < 0.001
217 parameters	Δρ_max_ = 0.41 e Å^−^^3^
0 restraints	Δρ_min_ = −0.53 e Å^−^^3^

### Special details

**Table:**

Geometry. All e.s.d.'s (except the e.s.d. in the dihedral angle between two l.s. planes) are estimated using the full covariance matrix. The cell e.s.d.'s are taken into account individually in the estimation of e.s.d.'s in distances, angles and torsion angles; correlations between e.s.d.'s in cell parameters are only used when they are defined by crystal symmetry. An approximate (isotropic) treatment of cell e.s.d.'s is used for estimating e.s.d.'s involving l.s. planes.
Refinement. Refinement of *F*^2^ against ALL reflections. The weighted *R*-factor *wR* and goodness of fit *S* are based on *F*^2^, conventional *R*-factors *R* are based on *F*, with *F* set to zero for negative *F*^2^. The threshold expression of *F*^2^ > σ(*F*^2^) is used only for calculating *R*-factors(gt) *etc*. and is not relevant to the choice of reflections for refinement. *R*-factors based on *F*^2^ are statistically about twice as large as those based on *F*, and *R*- factors based on ALL data will be even larger.

### Fractional atomic coordinates and isotropic or equivalent isotropic displacement parameters (Å^2^)

**Table:**

	*x*	*y*	*z*	*U*_iso_*/*U*_eq_	
P1	0.10931 (6)	0.10326 (17)	0.61793 (7)	0.0392 (4)	
O1	0.18993 (16)	0.1930 (4)	0.69493 (17)	0.0423 (7)	
O2	0.11788 (15)	0.2675 (4)	0.54921 (16)	0.0392 (7)	
O3	0.11527 (17)	−0.1319 (4)	0.6046 (2)	0.0498 (8)	
C1	0.2142 (2)	0.4112 (6)	0.6780 (2)	0.0356 (9)	
H1	0.1770	0.5201	0.6920	0.043*	
C2	0.1911 (2)	0.4166 (6)	0.5822 (2)	0.0364 (9)	
H2	0.1701	0.5677	0.5623	0.044*	
C3	0.0125 (2)	0.1775 (6)	0.6301 (2)	0.0372 (9)	
C4	−0.0305 (3)	0.0204 (6)	0.6596 (3)	0.0419 (10)	
H4	−0.0079	−0.1240	0.6713	0.050*	
C5	−0.1057 (3)	0.0729 (7)	0.6720 (3)	0.0441 (10)	
H5	−0.1338	−0.0340	0.6928	0.053*	
C6	−0.1395 (2)	0.2845 (7)	0.6535 (3)	0.0431 (10)	
H6	−0.1914	0.3209	0.6609	0.052*	
C7	−0.0975 (2)	0.4423 (7)	0.6243 (3)	0.0410 (9)	
H7	−0.1204	0.5864	0.6124	0.049*	
C8	−0.0221 (2)	0.3888 (6)	0.6125 (3)	0.0385 (9)	
H8	0.0062	0.4967	0.5923	0.046*	
C9	0.3069 (2)	0.4558 (6)	0.7331 (2)	0.0362 (9)	
C10	0.3694 (2)	0.2941 (7)	0.7478 (2)	0.0406 (9)	
H10	0.3546	0.1538	0.7227	0.049*	
C11	0.4540 (3)	0.3391 (7)	0.7996 (3)	0.0425 (10)	
H11	0.4967	0.2283	0.8100	0.051*	
C12	0.4766 (3)	0.5434 (7)	0.8360 (3)	0.0479 (11)	
H12	0.5343	0.5725	0.8714	0.057*	
C13	0.4146 (3)	0.7049 (7)	0.8205 (3)	0.0499 (11)	
H13	0.4298	0.8455	0.8452	0.060*	
C14	0.3300 (3)	0.6616 (6)	0.7686 (3)	0.0434 (10)	
H14	0.2878	0.7737	0.7575	0.052*	
C15	0.2596 (2)	0.3581 (6)	0.5482 (2)	0.0356 (9)	
C16	0.2620 (2)	0.1556 (7)	0.5115 (3)	0.0405 (9)	
H16	0.2230	0.0430	0.5121	0.049*	
C17	0.3218 (3)	0.1181 (7)	0.4740 (3)	0.0475 (11)	
H17	0.3228	−0.0195	0.4487	0.057*	
C18	0.3789 (3)	0.2794 (8)	0.4737 (3)	0.0513 (11)	
H18	0.4190	0.2531	0.4477	0.062*	
C19	0.3784 (3)	0.4806 (7)	0.5111 (3)	0.0494 (11)	
H19	0.4186	0.5910	0.5117	0.059*	
C20	0.3185 (2)	0.5192 (6)	0.5477 (3)	0.0416 (10)	
H20	0.3177	0.6575	0.5727	0.050*	

### Atomic displacement parameters (Å^2^)

**Table:**

	*U*^11^	*U*^22^	*U*^33^	*U*^12^	*U*^13^	*U*^23^
P1	0.0441 (7)	0.0296 (6)	0.0438 (7)	0.0021 (4)	0.0162 (5)	0.0008 (4)
O1	0.0442 (15)	0.0345 (15)	0.0459 (18)	−0.0013 (12)	0.0140 (13)	0.0093 (12)
O2	0.0440 (16)	0.0365 (15)	0.0369 (16)	−0.0033 (12)	0.0148 (12)	0.0003 (12)
O3	0.0563 (18)	0.0260 (15)	0.070 (2)	0.0028 (12)	0.0273 (16)	−0.0024 (13)
C1	0.050 (2)	0.0274 (19)	0.033 (2)	0.0038 (16)	0.0199 (18)	0.0019 (15)
C2	0.037 (2)	0.033 (2)	0.039 (2)	0.0013 (16)	0.0141 (17)	0.0017 (17)
C3	0.048 (2)	0.028 (2)	0.029 (2)	−0.0025 (17)	0.0077 (17)	−0.0065 (15)
C4	0.046 (2)	0.037 (2)	0.038 (2)	−0.0028 (18)	0.0108 (18)	0.0037 (17)
C5	0.053 (2)	0.043 (2)	0.037 (2)	−0.0083 (19)	0.0184 (19)	0.0030 (18)
C6	0.037 (2)	0.052 (3)	0.041 (3)	−0.0032 (18)	0.0157 (18)	−0.0034 (19)
C7	0.044 (2)	0.037 (2)	0.038 (2)	−0.0005 (18)	0.0103 (18)	−0.0017 (17)
C8	0.040 (2)	0.038 (2)	0.036 (2)	−0.0002 (17)	0.0114 (17)	0.0007 (17)
C9	0.045 (2)	0.036 (2)	0.029 (2)	0.0000 (17)	0.0162 (17)	−0.0018 (16)
C10	0.050 (2)	0.040 (2)	0.035 (2)	−0.0019 (18)	0.0189 (19)	−0.0010 (17)
C11	0.046 (2)	0.049 (3)	0.032 (2)	−0.0002 (19)	0.0150 (18)	0.0041 (18)
C12	0.050 (2)	0.054 (3)	0.036 (3)	−0.013 (2)	0.0117 (19)	−0.004 (2)
C13	0.063 (3)	0.042 (3)	0.048 (3)	−0.015 (2)	0.023 (2)	−0.010 (2)
C14	0.054 (3)	0.036 (2)	0.043 (3)	−0.0036 (18)	0.022 (2)	−0.0031 (18)
C15	0.044 (2)	0.034 (2)	0.029 (2)	0.0040 (17)	0.0139 (17)	0.0012 (15)
C16	0.042 (2)	0.041 (2)	0.037 (2)	0.0006 (17)	0.0127 (18)	−0.0045 (17)
C17	0.054 (3)	0.050 (3)	0.038 (3)	0.013 (2)	0.017 (2)	−0.0046 (19)
C18	0.052 (3)	0.066 (3)	0.040 (3)	0.008 (2)	0.021 (2)	0.004 (2)
C19	0.048 (2)	0.055 (3)	0.046 (3)	−0.002 (2)	0.018 (2)	0.009 (2)
C20	0.048 (2)	0.036 (2)	0.042 (3)	−0.0039 (18)	0.0185 (19)	0.0004 (17)

### Geometric parameters (Å, °)

**Table:**

P1—O3	1.462 (3)	C9—C14	1.388 (5)
P1—O1	1.600 (3)	C9—C10	1.392 (5)
P1—O2	1.601 (3)	C10—C11	1.393 (6)
P1—C3	1.769 (4)	C10—H10	0.9500
O1—C1	1.452 (4)	C11—C12	1.384 (6)
O2—C2	1.461 (4)	C11—H11	0.9500
C1—C9	1.514 (5)	C12—C13	1.383 (6)
C1—C2	1.556 (5)	C12—H12	0.9500
C1—H1	1.0000	C13—C14	1.392 (6)
C2—C15	1.515 (5)	C13—H13	0.9500
C2—H2	1.0000	C14—H14	0.9500
C3—C8	1.399 (5)	C15—C20	1.394 (5)
C3—C4	1.404 (5)	C15—C16	1.395 (5)
C4—C5	1.391 (6)	C16—C17	1.399 (6)
C4—H4	0.9500	C16—H16	0.9500
C5—C6	1.398 (6)	C17—C18	1.374 (6)
C5—H5	0.9500	C17—H17	0.9500
C6—C7	1.394 (6)	C18—C19	1.389 (6)
C6—H6	0.9500	C18—H18	0.9500
C7—C8	1.390 (6)	C19—C20	1.390 (6)
C7—H7	0.9500	C19—H19	0.9500
C8—H8	0.9500	C20—H20	0.9500
			
O3—P1—O1	112.38 (16)	C3—C8—H8	119.7
O3—P1—O2	117.82 (17)	C14—C9—C10	119.5 (4)
O1—P1—O2	97.11 (15)	C14—C9—C1	119.5 (3)
O3—P1—C3	112.89 (17)	C10—C9—C1	120.9 (3)
O1—P1—C3	109.87 (17)	C9—C10—C11	119.7 (4)
O2—P1—C3	105.51 (16)	C9—C10—H10	120.2
C1—O1—P1	111.0 (2)	C11—C10—H10	120.2
C2—O2—P1	113.1 (2)	C12—C11—C10	120.6 (4)
O1—C1—C9	109.6 (3)	C12—C11—H11	119.7
O1—C1—C2	104.9 (3)	C10—C11—H11	119.7
C9—C1—C2	117.3 (3)	C13—C12—C11	119.7 (4)
O1—C1—H1	108.3	C13—C12—H12	120.2
C9—C1—H1	108.3	C11—C12—H12	120.2
C2—C1—H1	108.3	C12—C13—C14	120.0 (4)
O2—C2—C15	110.4 (3)	C12—C13—H13	120.0
O2—C2—C1	104.1 (3)	C14—C13—H13	120.0
C15—C2—C1	119.0 (3)	C9—C14—C13	120.4 (4)
O2—C2—H2	107.6	C9—C14—H14	119.8
C15—C2—H2	107.6	C13—C14—H14	119.8
C1—C2—H2	107.6	C20—C15—C16	118.8 (4)
C8—C3—C4	118.8 (4)	C20—C15—C2	118.4 (3)
C8—C3—P1	122.2 (3)	C16—C15—C2	122.6 (3)
C4—C3—P1	119.0 (3)	C15—C16—C17	120.1 (4)
C5—C4—C3	121.0 (4)	C15—C16—H16	120.0
C5—C4—H4	119.5	C17—C16—H16	120.0
C3—C4—H4	119.5	C18—C17—C16	120.3 (4)
C4—C5—C6	119.4 (4)	C18—C17—H17	119.8
C4—C5—H5	120.3	C16—C17—H17	119.8
C6—C5—H5	120.3	C17—C18—C19	120.3 (4)
C7—C6—C5	120.3 (4)	C17—C18—H18	119.8
C7—C6—H6	119.9	C19—C18—H18	119.8
C5—C6—H6	119.9	C18—C19—C20	119.5 (4)
C8—C7—C6	120.0 (4)	C18—C19—H19	120.2
C8—C7—H7	120.0	C20—C19—H19	120.2
C6—C7—H7	120.0	C19—C20—C15	120.9 (4)
C7—C8—C3	120.6 (4)	C19—C20—H20	119.5
C7—C8—H8	119.7	C15—C20—H20	119.5
			
O3—P1—O1—C1	143.1 (2)	C4—C3—C8—C7	0.2 (6)
O2—P1—O1—C1	19.0 (2)	P1—C3—C8—C7	−178.8 (3)
C3—P1—O1—C1	−90.3 (3)	O1—C1—C9—C14	−139.9 (4)
O3—P1—O2—C2	−118.9 (3)	C2—C1—C9—C14	100.8 (4)
O1—P1—O2—C2	1.1 (2)	O1—C1—C9—C10	41.0 (5)
C3—P1—O2—C2	114.1 (2)	C2—C1—C9—C10	−78.4 (4)
P1—O1—C1—C9	−158.1 (2)	C14—C9—C10—C11	1.6 (6)
P1—O1—C1—C2	−31.4 (3)	C1—C9—C10—C11	−179.3 (3)
P1—O2—C2—C15	110.0 (3)	C9—C10—C11—C12	−0.5 (6)
P1—O2—C2—C1	−18.8 (3)	C10—C11—C12—C13	−0.3 (6)
O1—C1—C2—O2	30.1 (3)	C11—C12—C13—C14	0.1 (6)
C9—C1—C2—O2	152.0 (3)	C10—C9—C14—C13	−1.8 (6)
O1—C1—C2—C15	−93.2 (4)	C1—C9—C14—C13	179.1 (4)
C9—C1—C2—C15	28.6 (5)	C12—C13—C14—C9	1.0 (6)
O3—P1—C3—C8	−155.4 (3)	O2—C2—C15—C20	157.7 (3)
O1—P1—C3—C8	78.3 (4)	C1—C2—C15—C20	−82.1 (4)
O2—P1—C3—C8	−25.4 (4)	O2—C2—C15—C16	−17.2 (5)
O3—P1—C3—C4	25.5 (4)	C1—C2—C15—C16	103.0 (4)
O1—P1—C3—C4	−100.8 (3)	C20—C15—C16—C17	−1.0 (6)
O2—P1—C3—C4	155.5 (3)	C2—C15—C16—C17	173.9 (4)
C8—C3—C4—C5	−0.6 (6)	C15—C16—C17—C18	0.6 (6)
P1—C3—C4—C5	178.5 (3)	C16—C17—C18—C19	0.5 (7)
C3—C4—C5—C6	1.0 (6)	C17—C18—C19—C20	−1.2 (6)
C4—C5—C6—C7	−1.1 (6)	C18—C19—C20—C15	0.7 (6)
C5—C6—C7—C8	0.7 (6)	C16—C15—C20—C19	0.4 (6)
C6—C7—C8—C3	−0.3 (6)	C2—C15—C20—C19	−174.8 (4)

### Hydrogen-bond geometry (Å, °)

**Table:**

Cg1 is the centroid of the C15–C20 ring.

**Table:**

*D*—H···*A*	*D*—H	H···*A*	*D*···*A*	*D*—H···*A*
C1—H1···O3^i^	1.00	2.59	3.248 (5)	123 (3)
C2—H2···O3^i^	1.00	2.29	3.115 (5)	139 (3)
C12—H12···Cg1^ii^	0.95	2.94	3.838 (5)	158 (3)
C12—H12···C18^ii^	0.95	2.83	3.585 (6)	138 (3)

Symmetry codes: (i) *x*, *y*+1, *z*; (ii) −*x*+1, *y*+1/2, −*z*+3/2.
